# Temporal Changes in Randomness of Bird Communities across Central Europe

**DOI:** 10.1371/journal.pone.0112347

**Published:** 2014-11-11

**Authors:** Swen C. Renner, Martin M. Gossner, Tiemo Kahl, Elisabeth K. V. Kalko, Wolfgang W. Weisser, Markus Fischer, Eric Allan

**Affiliations:** 1 Institute of Experimental Ecology, University of Ulm, Ulm, Germany; 2 Smithsonian Conservation Biology Institute, National Zoological Park, Front Royal, Virginia, United States of America; 3 Terrestrial Ecology Research Group, Department of Ecology and Ecosystem Management, Centre for Food and Life Sciences Weihenstephan, Technische Universität München, Freising, Germany; 4 Chair of Silviculture, University of Freiburg, Freiburg, Germany; 5 Institute of Plant Sciences and Botanical Garden, University of Bern, Bern, Switzerland; 6 Institute of Plant Sciences, University of Bern, Bern, Switzerland; Institute of Agronomy, University of Lisbon, Portugal

## Abstract

Many studies have examined whether communities are structured by random or deterministic processes, and both are likely to play a role, but relatively few studies have attempted to quantify the degree of randomness in species composition. We quantified, for the first time, the degree of randomness in forest bird communities based on an analysis of spatial autocorrelation in three regions of Germany. The compositional dissimilarity between pairs of forest patches was regressed against the distance between them. We then calculated the *y-*intercept of the curve, i.e. the ‘nugget’, which represents the compositional dissimilarity at zero spatial distance. We therefore assume, following similar work on plant communities, that this represents the degree of randomness in species composition. We then analysed how the degree of randomness in community composition varied over time and with forest management intensity, which we expected to reduce the importance of random processes by increasing the strength of environmental drivers. We found that a high portion of the bird community composition could be explained by chance (overall mean of 0.63), implying that most of the variation in local bird community composition is driven by stochastic processes. Forest management intensity did not consistently affect the mean degree of randomness in community composition, perhaps because the bird communities were relatively insensitive to management intensity. We found a high temporal variation in the degree of randomness, which may indicate temporal variation in assembly processes and in the importance of key environmental drivers. We conclude that the degree of randomness in community composition should be considered in bird community studies, and the high values we find may indicate that bird community composition is relatively hard to predict at the regional scale.

## Introduction

Understanding the processes determining the species richness, diversity, and abundance of organisms remains a key challenge. Deterministic processes such as those driven by habitat structure and heterogeneity [1]–[3], species-specific ecological traits [4], [5], seasonality [6], or resource availability such as food [7], have been shown to be important in many ecosystems. Stochastic processes such as neutral dynamics [8] may, however, also explain a proportion of the species richness and diversity of communities [9]. As both types of processes are likely to be important in driving community assembly it is crucial to determine their relative importance and to understand to what extent stochastic processes shape the community structure of organisms. If random processes play a significant role, predicting changes in community composition may be challenging.

Among animals, birds are ecologically well-known and easy to identify and thus form part of many ecological studies. Many studies have explored the drivers of bird community composition, including land management intensification, climate change [10], alterations in habitat structural parameters [1], [2], and changes in resource availability [7], or nest site availability [11]. However the role of stochastic processes in affecting bird communities has seldom been explored or quantified [12].

Land use intensification has resulted in substantial declines in bird diversity [5], [10], [13]. In particular, forest conversion from mainly hardwood to softwood species has resulted in declines of many bird species in areas with high human population pressure [6]. Land use intensification is also likely to alter assembly processes and therefore change the relative importance of deterministic and stochastic processes in driving community composition. An increase in management intensity might be expected to increase the importance of deterministic processes because only species adapted to high land use intensity can persist. This would imply that environmental filters play a larger role, and community composition would be expected to become more homogenous, in these landscapes. Moreover, high land use intensity is likely to reduce redundancy [14] which might also decrease the importance of random processes in driving community composition. We would therefore expect a higher degree of randomness in community composition at low management intensity than at high management intensity. Some studies have suggested that randomness is reduced in more disturbed sites [15]–[17], but the relative importance of deterministic and random processes in shaping communities under different land use intensities has not been studied. Moreover it is unclear whether these effects are consistent across differently managed forest habitats.

Community composition may turn over substantially between years and the importance of different assembly processes may also change over time [18]. Long-term studies on birds have shown that relative abundance, species composition and species diversity can vary considerably between years [12]. Factors driving species turnover between years include extreme weather events, habitat fragmentation [19], or population processes such as immigration, dispersal, or mortality [20]. If the strength of these processes, in determining species composition, varies over time, then the importance of stochastic processes might also vary over time. However, how the importance of random processes varies over time has not been quantified.

Calculating the proportion of community composition that is determined by random processes is challenging. The traditional approach is to use a null model to determine the deviation of observed composition from that expected by chance. Creating a null model is, however, technically not trivial and in some cases may even be impossible [9], [17]. An alternative approach was proposed by Brownstein et al. [17], who suggested using the *y-*intercept (the ‘nugget’) from a regression of community similarity against spatial distance as a measure of the proportion of the community composition determined by random processes. Differences in species composition between sites will be due to spatially autocorrelated environmental differences, dispersal limitation and chance. The effects of chance are expected to be the same across a spatial gradient, so that at zero spatial distance only the effects of chance remain to drive differences in species composition. The nugget is conceptually the dissimilarity in species composition at zero geographic distance, which would be expected to be zero if geographic distance explained the variation in species composition, i.e. if there was no influence of stochastic processes. Values of the nugget greater than zero can therefore be interpreted as indicating the influence of random or chance processes, with larger nuggets indicating a larger role for random processes in determining community composition. We use the terms ‘chance’ and ‘randomness’ synonymously to refer to those processes which result in community compositions that are unpredictable from the (spatially autocorrelated), biotic and abiotic environment.

We analyse the relative strength of random processes in driving bird community assembly across local and regional scales and across time. We study bird communities in three regions of Germany, in forests varying in management intensity, across five consecutive years (2008 to 2012). We hypothesise that random processes will be important in driving bird community composition and will vary over time but their importance will be reduced under intensive forest management. We also hypothesise that the degree of randomness found in bird communities is not “stable” and in fact varies substantially over time.

## Materials and Methods

### Study regions and sites

Our study is part of the large-scale and long-term research platform ‘Biodiversity Exploratories’ (a detailed description of the study area, selection of study regions and sites and classification procedures is given in [21]).

**Figure 1:**
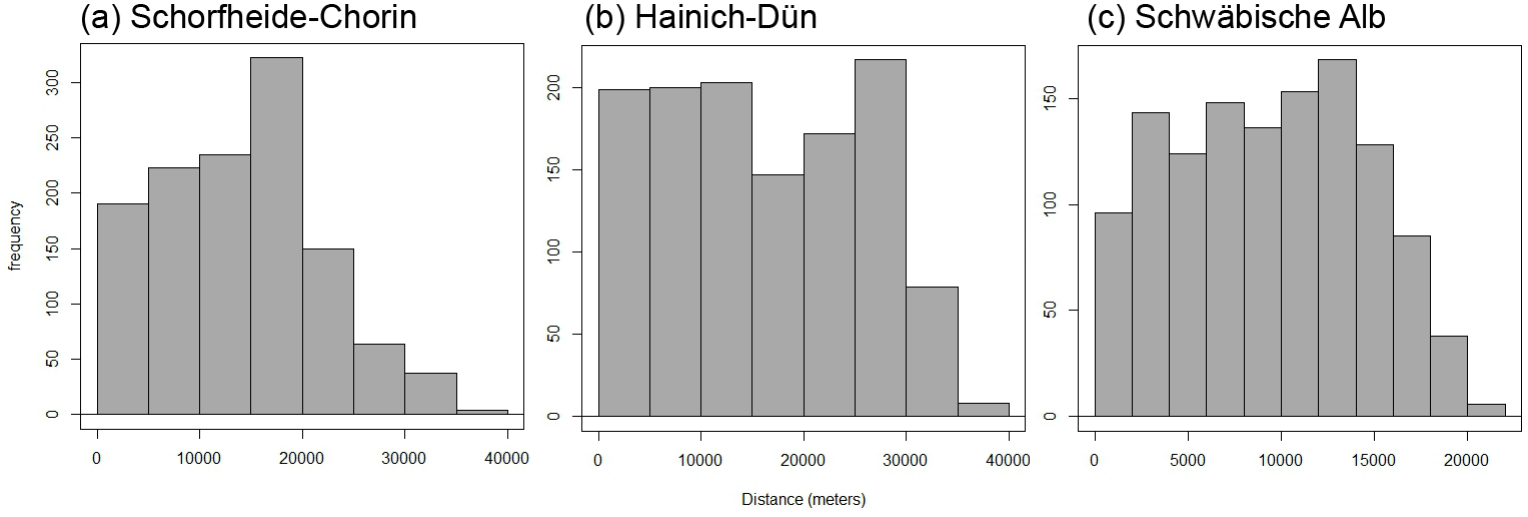
Histograms of Euclidian distance in meters between all 1225 possible distance of forest sites for each of the three regions. Note the different scales in each histogram.

**Figure 2:**
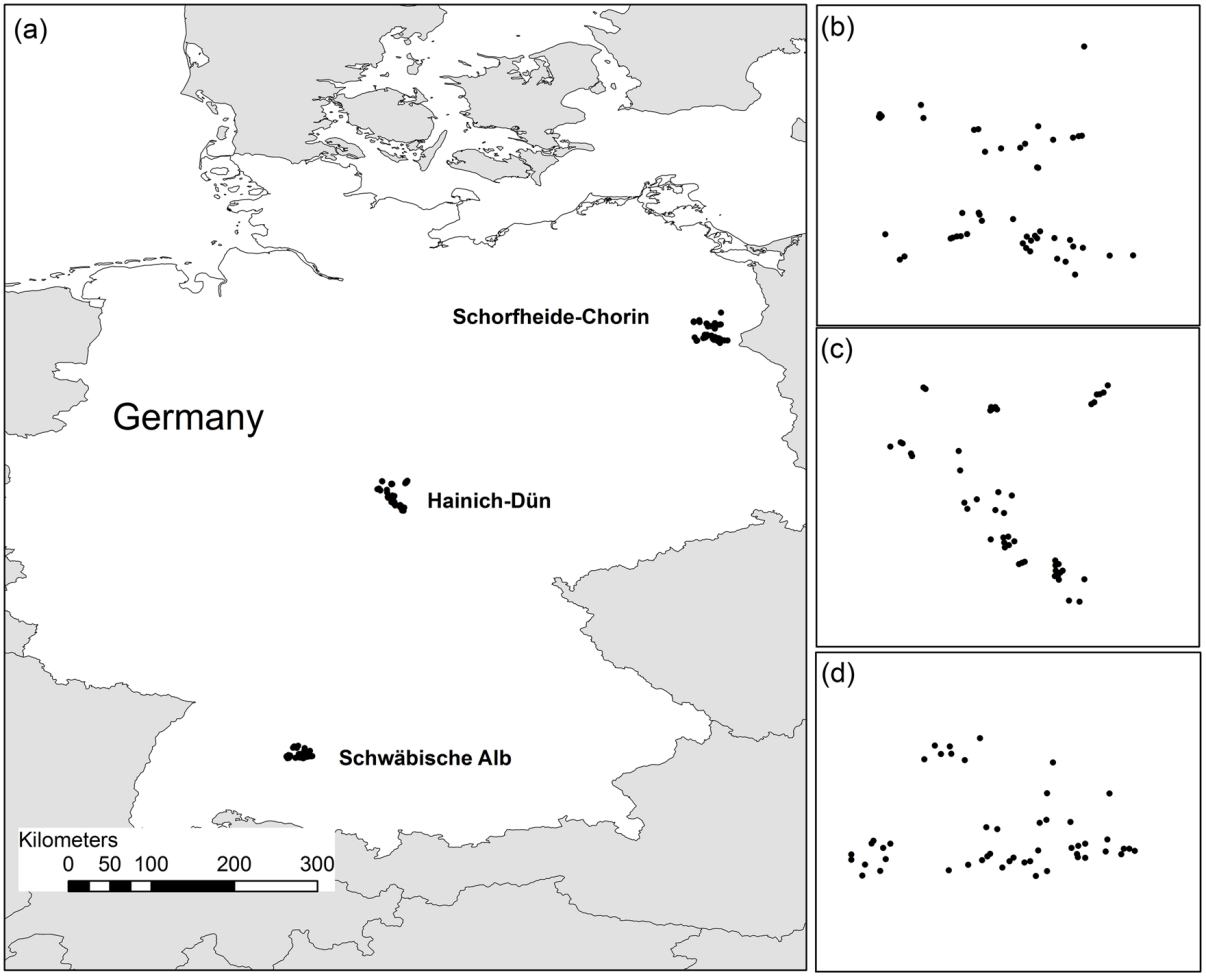
Spatial distribution of all 150 sites across the three regions. (**a**) Locations of the three regions within Germany and distribution of the sites within Schorfheide-Chorin (**b**), Hainich-Dün (**c**), and Schwäbische Alb (**d**).

In total, we studied 150 forest plots in three regions of Germany: the south-west region (Schwäbische Alb; approximate centre coordinates: 48.4° North, 9.5°East, altitude 500 to 800 m a.s.l.), the central region (Hainich-Dün; 51.1° North, 10.4°East, 285 to 550 m), and the north-east region (Schorfheide-Chorin; 53.0° North, 13.9°East, 3 to 140 m). Each plot was covered by forest which was homogenous in terms of canopy tree species composition, soil, and mean slope ≤20% [21]. Forest plots were between 250 m and 45 km apart within each of the three regions (Figure 1), which means we estimate effects at small spatial scales. A large number of small spatial distances are necessary to provide a reliable estimate of the nugget [17]. The lack of larger spatial distances (>100 km) is problematic if the dissimilarity values do not asymptote or do not reach 1, and if there is substantial dispersal limitation. Therefore we did not analyse the nuggets between the three regions. Plots within each region span a large gradient in management intensity and cover a large area (Figure 2).

### Land use intensification

To understand whether the degree of randomness in community composition is linked to forest management we split all forest plots per region into whether they were managed at high or low intensity. We used a compound land use intensity index (details outlined in Appendix S1; [22]), which is based on the harvested tree volume, abundance of coniferous trees (as an indicator of “naturalness”, conifers are not part of the natural forest in these study areas) and volume of dead wood with saw cuts (as an indicator of disturbance). Using this index, we divided all plots per region into the 25 with management intensity higher than the median and the 25 with management intensity lower than the median.

### Bird surveys

At each of the 150 sites we surveyed birds by standardized audio-visual point-counts and recorded all birds exhibiting territorial displays (singing and calling activity) for five minutes per point count locality and time period. We used 50-m-fixed-radius point counts and noted all males of each bird species during the five-minute interval. Each site was visited five times between 15 March and 15 June (first surveying period 15–30 March; 2^nd^ 15–30 April; 3^rd^ 1–15 May; 4^th^ 16–31 May; 5^th^ 1–15 June) in 2008 to 2012.

A minimum of five and a maximum of 15 sites were surveyed per day by one observer from sunrise to 11∶00 h; occasionally the evening chorus was surveyed after 17∶00 h to sunset (<20 times out of 750 events per year). The sequence in which sites were visited was randomized. Each song or call heard on a site was interpreted as one male territorial display behaviour. The maximum number of birds displaying site^−^^1^ year^−^^1^ (i.e. the maximum number of individuals per species observed in any of the five surveys) was used as a measure of the relative abundance of each bird species. We considered a species as present in any given site if it was recorded at least once during a survey round within any given year. Aerial species (swifts and swallows) were excluded from analysis, since they had been surveyed irregularly and are biased towards beech forests (where detectability for aerial species is higher than in spruce forests).

The data is accessible through the Biodiversity Exploratories database http://www.biodiversity-exploratories.de/intranet/ (follow the link “BExIS”; registration required).

### Data analyses

First we used generalized linear mixed effect models (GLMM) to test whether bird species richness or relative abundance was affected by time, region, site, or management intensity and the interaction between these factors. The model was specified as: species richness ∼year * region * management intensity + (year | site id). The model was fit with Poisson errors. In a second model, we analysed the response of relative abundance instead of species richness.

We calculated the relative degree of randomness in bird community composition following Brownstein et al. [17]. This approach calculates the nugget from the relationship between dissimilarity in species composition and geographic distance and uses this as a measure of the proportion of the species composition explained by chance. This is conceptually the dissimilarity in species composition at zero geographic distance, which would be expected to be zero if geographic distance explained the variation in species composition, i.e. if there was no influence of stochastic processes. We calculated dissimilarity in bird species composition between sites using the Jaccard index *D*’, with EstimateS 8.2 [23], and did this separately for each of the three regions (north-east, central, and south-west).

We calculated the nuggets for each year separately and across years. For the analysis across years we used all of the species observed per site across the five years and used this cumulative species list to calculate dissimilarity. We then determined the distance in meters between all sites within each of the three regions (Euclidian distance from each site centroid to site centroid).

**Figure 3:**
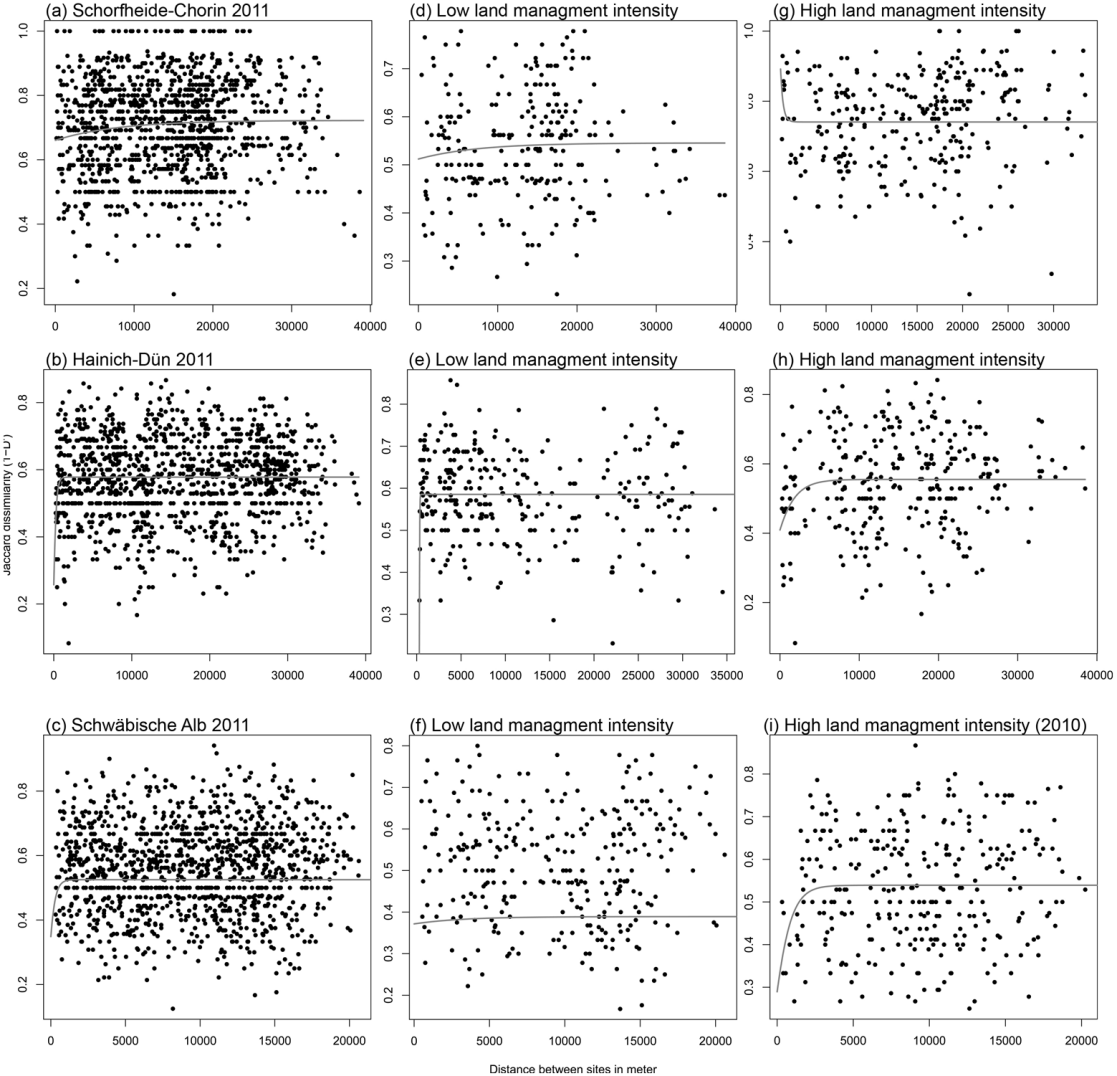
Dissimogram for the bird communities showing the Jaccard dissimilarity between each possible site pair *versus* Euclidian distance between the forests sites. (**a–c**) all sites in 2011, (**d–f**) sites with low land use intensity, and (**g–i**) sites with high land use intensity. The *x*-axis is the distance between each pair of sites. The *grey line* is the fitted line estimated based on non-linear least squares. Note the different scales in each *x*-axis.

Dissimilarities in species composition between all possible 1,225 pairs of experimental sites per region were related to the geographic distance between them. A non-linear least squares equation was used to model the relationship between the Jaccard *D*’ dissimilarities (1-*D*’), as the *y-*variable, and spatial distance. We therefore fitted a spatial autocorrelation dissimogram (Figure 3) [24]: *D*’*_spatial_* = *a* • e ∧ (–*b* • e ∧(–*c* • *d*)), where *a*, *b* and *c* are fitted parameters estimated with the Gauss-Newton algorithm, and *d* is distance between plots. We used package nls2 [25] in R [26] to fit all of the models.

We used different algorithms to explore the suitability of different equations for calculating the nugget [17]. Only two formulae resulted in models which converged (Gompertz and Negative exponential), all others resulted in many fewer models converging (Appendix S2). From the fitted curves (examples in Figure 3) we determined the nugget (*a* • e ∧ –*b*) as the *y-*intercept and the Asymptote (*a*), which represents the dissimilarity at infinite distance (i.e., the fitted maximum dissimilarity).

Nuggets >1.0 were excluded from the analysis, as they indicate poorly fitting models. We also calculated the amount of variance explained by each of the models, using a pseudo *R* ^2^. We calculated this as the square of the Pearson correlation coefficient for the correlation between model fitted values and the original data. If the pseudo *R* ^2^ is low then a small amount of the variation in community composition is explained by geographic distance. In general, pseudo *R* ^2^ approaches may not be entirely appropriate for non-linear models but they do convey an idea of the goodness-of-fit.

### Further statistical analysis

To understand whether species richness and stochasticity are related, we assessed associations of species richness and the nuggets using a linear model in R (R command lm).

Detectability and occupancy of sites by bird species might affect our analysis: low detectability of species might bias our results by increasing variation in species composition between sites and therefore increasing our estimate of the degree of randomness in species composition. Species cannot always be detected even when they are present at a site but repeated surveys (typically ≥3 repetitions) at a given site reduce this detection bias [27], [28]. To further assess whether our data is biased through detection probability, we calculated the detectability (estimate of ψ; [27]) of each bird species in each plot to determine if low detectability could have an influence on our estimate of the degree of randomness in community composition. We applied the “multi-season” model in PRESENCE 6.1 [28] and calculated overall detectability for each species over five years and five repetitions within each year.

In addition to calculating the detectability of individual species, we calculated the inter-annual turnover in species composition for each plot. We might expect that if the nuggets are driven by measurement error, i.e. high nuggets are due to the fact that we have failed to completely sample the local bird community, then excluding plots with high turnover values will reduce the size of the nuggets. We first determined for each plot all the species seen in any of the five years (i.e. the cumulative species richness) and then the species which were seen across the whole five-year period (i.e. those observed in four or five years in the plot). We then calculated the turnover between the species seen in ≥4 years and those seen in <4 years for each plot. Finally, we repeated our calculations of the nuggets, excluding those plots with turnover values of 60%, 70%, or 80%.

## Results

**Figure 4:**
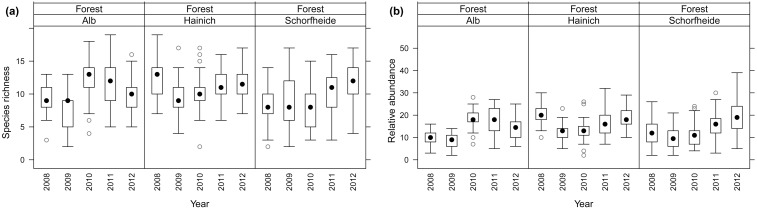
Temporal variation in the bird communities across five consecutive years (2008–2012) in the three study regions. (**a**) Temporal variation in species richness (counted bird species), and (**b**) temporal variation in relative abundance. The *Black dot* represents the median, the *Box* 1^st^ and 3^rd^ quartile, *whiskers* 95% confidence intervals and *grey circles* outliers.

In total, we observed 82 bird species in the three regions over the five consecutive years. The species richness of birds varied considerably between the three regions and across time (Figure 4a). Species richness was significantly lower in the south-west region compared to the other two regions (GLMM: *p*≤0.01; Figure 4a; detailed information in Appendix S3), and management intensity decreased species richness. The relative abundance of birds in the three regions showed a similar pattern to species richness with significant differences between years and the regions and also lower abundance in the south-west (*p*≤0.02). Management intensity reduced abundance (*p*≤0.02). In general, inter-annual variation was higher than the between region variation for both species richness and relative abundance.

### Degree of randomness in bird community composition

A large proportion of bird community composition was explained by random processes (Table 1, example in Figure 3). Using the cumulative species richness per site, the nuggets from the dissimogram ranged between 0.25 and 0.86 (Gompertz equation). This suggests that random factors alone cause high turnover between communities.

**Table 1:** Summary of estimated nuggets for the bird communities from 2008 to 2012.

Year(s)	Gompertz	Negative Exponential	High land use intensity	Low land use intensity	60% cut-off^b^	70% cut-off^b^	80% cut-off^b^
Cumulative species overyears Schorfheide-Chorin (north-east)2008–2012^a^	0.6660	0.6689	n/a	0.5323	0.6073	n/a	n/a
2008	0.8603	n/a	n/a	0.9336			
2009	0.6606	0.6603	n/a	0.3285			
2010	0.6604	0.6604	0.7511	0.5310			
2011	0.6604	0.6604	n/a	0.5120			
2012	n/a	n/a	n/a	0.0860			
Mean (2008–2012)	0.7104	0.6604	0.7511	0.4782			
± s. d. (2008–2012)	0.0999	0.0001	n/a	0.3113			
Minimum (2008–2012)	0.6604	0.6603	0.7511	0.0860			
Maximum (2008–2012)	0.8603	0.6604	0.7511	0.9336			
Cumulative species overyears Hainich-Dün(centre) 2008–2012^a^	0.5077	0.5072	0.4910	0.5182	0.3983	0.4859	0.5093
2008	0.4430	0.4424	0.4511	0.4093			
2009	0.5390	0.5376	n/a	0.5554			
2010	0.5543	0.5547	n/a	n/a			
2011	0.2573	0.1950	0.4093	0.0000			
2012	0.4474	0.4463	0.2481	n/a			
Mean (2008–2012)	0.4482	0.4352	0.3695	0.3216			
± s. d. (2008–2012)	0.1183	0.1437	0.1072	0.2879			
Minimum (2008–2012)	0.2573	0.1950	0.2481	0.0000			
Maximum (2008–2012)	0.5543	0.5547	0.4511	0.5554			
Cumulative species overyears SchwäbischeAlb (south-west)2008–20	n/a	0.5384	0.4416	n/a	0.4945	0.2243	n/a
2008	0.4369	0.4365	0.3487	0.3715			
2009	n/a	n/a	n/a	n/a			
2010	0.3486	0.3351	0.2894	n/a			
2011	n/a	0.1300	n/a	n/a			
2012	0.6261	0.6261	0.5329	0.5535			
Mean (2008–2012)	0.4705	0.3819	0.3903	0.4625			
± s. d. (2008–2012)	0.1418	0.2068	0.1270	0.1287			
Minimum (2008–2012)	0.3486	0.1300	0.2894	0.3715			
Maximum (2008–2012)	0.6261	0.6261	0.5329	0.5535			

“n/a” indicates that the model did not converge or that the values for nuggets were >1 or <0.

a Calculated from Jaccard Dissimilarity (1-*D*’) calculated with the cumulative species per plot over five years, i.e. all of the species observed at least once on the plot during this time period.

b The analysis was restricted to those plots with low inter-annual species turnover to determine if this influenced the high nuggets. Plots with turnover values higher than 60%, 70%, or 80% were excluded, see methods for details on the calculation of turnover.

In contrast to our hypothesis we found substantial variation in the degree of randomness in bird communities within the same site across years. Over three years (2010 to 2012), during which the observers and effort were constant, the nuggets calculated varied between 0.393 and 0.763 in the central region (Hainich-Dün), from 0.859 to 0.926 in the south-west (Schwäbische Alb) and from 0.689 to 0.808 in the north-east (Schorfheide-Chorin) (Table 1). The 95% confidence intervals and the lower and upper limits of the mean nuggets per forest sites over five years indicate significant temporal variation (Table 2).

**Table 2:** Confidence Intervals (CI, 95%) with lower and upper limit of CI for mean nuggets over the five consecutive years.

Region	Mean	CI	lower	upper
Schorfheide-Chorin (north-east)	0.710	0.138	0.573	0.848
Hainich-Dün (centre)	0.448	0.131	0.317	0.580
Schwäbische Alb (south-west)	0.471	0.288	0.183	0.758

Management intensity did not affect the degree of randomness in community composition (Table 1). However, the variation in the nugget over time was somewhat higher at low management intensity (0 to 0.93), than at high management intensity (0.24 to 0.75) (Table 1). Bird species richness and abundance were not related to the nugget (linear model: species richness Adjusted *R* ^2^ = −0.083, F = 0.001, *P* = 0.974, abundance Adjusted *R* ^2^ = −0.004, F = 0.942, *P* = 0.351). We therefore did not find any consistent changes in the nugget based on diversity or land use intensification.

Non-linear least square model-fit was relatively low and the highest pseudo *R* ^2^ value observed was 13.4% (Table 3). Therefore, space explained only a small portion of the variation in species richness. However, the nuggets and pseudo *R* ^2^ values were not correlated (linear model; F = 0.440, *P* = 0.528), indicating that low pseudo *R* ^2^ are not the only reason for the high nuggets that we found. Differences between the converging models with Gompertz or Negative Exponential functions were negligible and the nuggets calculated by the two methods diverged by less than 1% from each other (except in three cases where they diverged by 11%, 6%, and 3%). This indicates that our results were not sensitive to the particular function used to model the relationship between distance and dissimilarity.

**Table 3:** Pseudo *R* ^2^ between original and fitted values in non-linear least square analysis.

Year(s)	Allforest	High land-use intensity	Low land-use intensity	60%Cut-off^b^	70%Cut-off^b^	80%Cut-off^b^
Schorfheide-Chorin (north-east) 2008–2012^a^	0.016	0.000	0.019	0.091	n/a	0.006
2008	0.000	0.002	0.000			
2009	0.011	0.004	0.010			
2010	0.010	0.000	0.048			
2011	0.010	0.000	0.005			
2012	0.008	0.000	0.005			
Hainich-Dün (centre) 2008–2012^a^	0.130	0.069	0.071	0.121	0.093	0.129
2008	0.072	0.027	0.050			
2009	0.054	n/a	0.063			
2010	0.056	0.003	0.006			
2011	0.007	0.030	0.007			
2012	0.043	0.085	0.099			
Schwäbische Alb (south-west) 2008–2012^a^	n/a	0.022	n/a	0.018	0.002	n/a
2008	0.006	0.016	0.000			
2009	n/a	0.003	n/a			
2010	0.001	0.019	n/a			
2011	n/a	n/a	0.000			
2012	0.005	0.020	0.004			

a Calculated from Jaccard Dissimilarity (1-*D*’) calculated with the cumulative species per plot over five years, i.e. all of the species observed at least once on the plot during this time period.

b The analysis was restricted to those plots with low inter-annual species turnover to determine if this influenced the high nuggets. Plots with turnover values higher than 60%, 70%, or 80% were excluded, see methods for details on the calculation of turnover.

### Detectability and plot-based species turnover

The mean detectability of the 82 bird species in the 150 forest sites was ψ = 0.57 (i.e. on average, 57% of individuals per species were detected). This level of detectability is relatively high, indicating that our sampling was fairly complete. To further assess the influence of sampling on the nuggets we repeated our analysis excluding those plots which experienced high temporal turnover in species composition. This made little difference to the nuggets, which remained high even when plots with high species turnover were excluded (Table 1): the confidence intervals of the nuggets with high turnover plots included those calculated by all other analysis (compare Table 2).

## Discussion

Our analyses suggested that random processes were important in structuring our forest bird communities, with around half of the variation in species composition between communities explained by chance. This might suggest an important role for stochastic processes in affecting bird community structure [9], [17], [29]–[31]. Our analysis assumes that the major environmental factors driving bird community composition are spatially autocorrelated. This is likely to be true for factors such as climate and weather [6], [10] but might not be the case for land use [1], [2], [6], forest structure [2] or food and nest site availability [7]. In addition, the approach by Brownstein et al. [17] has limitations: on the one hand the approach will work only with small spatial distances (<100 km), otherwise the distance decay and dispersal limitations will overrule the observable effects at these small distances. On the other hand, at very small spatial extent (“initial similarity” at <1 km) other ecological factors such as extent, latitude, or body size might add up towards random factors [32]. Therefore distances between 1 and 100 km are best suited for calculating the nugget. However, with these caveats in mind, our analysis does suggest that a large fraction of bird community composition is not predictable from the environment.

### Effect of spatial extent and dispersal ability

The values for the degree of randomness in community composition which we found were higher than those found by the only other study to quantify the degree of randomness in species composition, using this particular method. Brownstein et al. [17] found less randomness in community composition in sessile plant communities. This difference in the degree of randomness found in the two studies might relate to the different dispersal abilities of the two groups of organism. In plants, dispersal is much more limited than for birds [33]. Even sedentary bird species can have flexible home ranges and do not typically remain in a fixed area [34,35 36]. Birds can therefore easily disperse between forest sites and the foraging range of even medium-sized passerines is typically <2 ha and in rare cases it can exceed 10 ha [37]. Bird species may therefore have been widely distributed within the three regions, meaning that even geographically distant plots could still have a similar bird community composition. The low pseudo *R* ^2^ values we found indicate that spatial distance might not explain much of the variation in species composition and this might have led to less reliable estimates of the nugget. Including more distant plots in the analysis might have led to a better relationship between space and compositional turnover. However, Brownstein et al. [17] also found that distance explained little variance in dissimilarity in their plant communities (1.2% to 23%), so this does not seem to be the major cause of the difference from our results. Future studies analysing bird communities at larger spatial extents are necessary to see if the high degree of randomness we find is a result of the spatial extent of our study. Our analysis nevertheless suggests that, at least at this spatial scale, a substantial degree of variation in bird community composition is not predictable from the environment.

### Effects of species turnover and detectability

As well as being affected by the degree of randomness in community composition, the large nuggets we found could also be driven by observer bias and low detectability of species. Even well trained and experienced ornithologists can miss up to 10% of bird species during surveys [38], [39]. While such errors might play a role in our results they cannot explain the high temporal variation because the same observers carried out all surveys in the central region and four out of five years in the north-eastern region. The spatial grain of our sampling might also play a role and larger plots might have resulted in more predictable species composition because rare species would be more likely to be missed in small plots. However the opposite is also possible if large plots contain more microhabitats and therefore have more variable species compositions [17]. We were not able to test for the effect of spatial grain here and further studies are needed to determine the effect of spatial grain on the predictability of bird community composition. To better assess the issue of detection probabilities, we calculated detectability ψ for all of the bird species and found that our mean detection probabilities (ψ = 0.57) were comparatively high compared to some other studies on bird communities, with mean detectability of 0.15≤ ψ ≤0.43 [40], [41]. Issues in detectability are typically reduced by increasing number of repetitions [27], [28], where each repetition increases the chance that the bird community is sampled completely (or at least more completely). Because we have a comparatively high number of repetitions per site, we assume that an even higher effort to repeat surveys would not decrease the degree of randomness (cf. temporal variation below). In addition, excluding plots with high levels of species turnover across time from our analysis did not significantly reduce the nuggets, which would be expected if incomplete sampling of species drove the high nuggets. These results indicate that sampling error is not the only factor driving our estimates for the large degree of randomness in species composition. Our results do therefore suggest that a substantial fraction of the variation in local bird community composition is driven by random processes.

### Temporal and spatial variation

The degree of randomness in bird community composition varied substantially between years. We also found temporal variation in the species richness and abundance of the bird communities, which suggests that there was high turnover in bird community composition between years. Other studies have shown spatial variation in the degree of randomness [17], but temporal variation has not been quantified so far. Temporal variation in the degree of randomness might arise because the deterministic drivers of bird community composition, such as weather or seasonality in resource availability can vary considerable between years. The degree of randomness in community composition would vary between years if the strength of these deterministic drivers also varies across time, i.e. so that climate strongly determines bird community composition in some years but has a relatively weak effect on community composition in other years. In the years in which it has a weak effect, composition might vary more stochastically between communities. Increasing detectability (by increasing the number of repetitions [27], [28]), could in theory reduce this temporal variation in the degree of randomness. Each additional repetition increases the chance that the bird community is more completely sampled. We have a comparatively high number of repetitions per site and per year and a therefore comparatively high ψ. Therefore a more exhaustive sampling protocol, with even more frequent sampling per year, would be unlikely to reduce the temporal variation we found. The high temporal variation in the degree of randomness indicates that future studies need to more often consider temporal variation in the drivers of community composition.

We also found spatial variation and large differences in the degree of randomness between our three regions. This agrees with other studies, which have observed spatial variation in randomness [17], [38]. The three regions varied in both species numbers and relative abundance of bird species and this may have caused the variation in degree of randomness between the regions. These results show that conclusions about the importance of random processes in driving community composition should be based on wide spatial and temporal sampling.

### Effects of land use intensity

Management intensity did not have an effect on the nugget, i.e. our results suggest that increased land use intensification does affect the degree of randomness in community composition. We expected that high management intensity would reduce the influence of random processes in structuring bird communities, however we did not find evidence for this. Management intensity did, however, affect both species richness and abundance of birds, which indicates that it is an important driver of bird communities. However it does not seem to alter the degree of randomness, which remains high even in more intensively managed forests.

### Conclusion

The Brownstein model provides a simple method for calculating the degree of randomness in community composition from spatially explicit data. Using this method we find that a large proportion of the variation in bird community composition between sites is driven by random processes. This means that bird community composition may not be predictable from the environment alone, at least at this spatial scale, and that predicting shifts in local bird community composition in response to global change may be difficult. If stochastic processes do play a large role in determining bird community composition, it also means that birds may not be ideally suited as indicators of diversity for other groups of organisms. We conclude that determining the degree of randomness is important for analyses of community structure, particularly as we suggest that random processes may play a surprisingly large role in driving community composition.

## Supporting Information

Figure S1 **Bird species numbers (** ***dots*** **) and abundance (** ***circles*** **) in relation to land use intensity (** ***W_i_*** **) in forest sites over all three regions.**
(TIF)

Appendix S1 **Explanations of management intensity index.**
(DOCX)

Appendix S2 **Formula used to calculate the “nugget”.**
(DOCX)

Appendix S3 **Summary statistics of GLMM results.**
(DOCX)

Appendix S4 **Relevant data as used for nls() modelling.**
(XLSX)
