# Supplementary figures and images for: Temporal Changes in Randomness of Bird Communities across Central Europe
Source: PLoS One. 2014 Nov 11;9(11):e112347. doi: 10.1371/journal.pone.0112347 (PMC4227846; doi:10.1371/journal.pone.0112347)

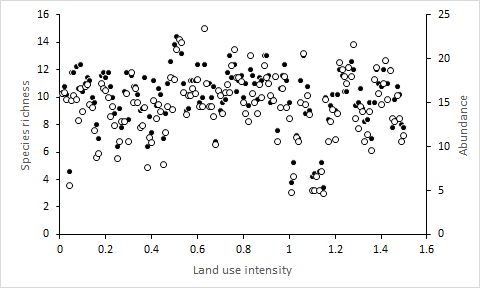

Supplement: Figure S1 — Bird species numbers ( dots ) and abundance ( circles ) in relation to land use intensity ( Wi ) in forest sites over all three regions. (TIF) [file pone.0112347.s001.tif]
