# Supplementary material for: Temporal Changes in Randomness of Bird Communities across Central Europe
Source: PLoS One. 2014 Nov 11;9(11):e112347. doi: 10.1371/journal.pone.0112347 (PMC4227846; doi:10.1371/journal.pone.0112347)
Supplement: Appendix S1 — Explanations of management intensity index. (DOCX) [file pone.0112347.s002.docx]

Appendix S1. Explanations of management intensity index.

Renner, Gossner, Kahl, Kalko, Weisser et al.

The land management intensity index for forests uses coniferous stock portion, harvested stock and dead wood in stock (Kahl and Bauhus 2014 [22]). For each site (plot) *i*, the land-use *W_i_* is defined as the sum of each variable:

*W_i_ = I_conif_ + I_harv_ + I_dwcut_*

where *I_conif_* is the proportion of coniferous stock (harvested + standing), *I_harv_* is the proportion of harvested stock, and *I_dwcut_* is the proportion of dead wood showing chain saw cuts. In relation to bird abundance and species numbers (Figure S1), the forest land use intensity index shows variation but no trend.
