# Supplementary material for: Temporal Changes in Randomness of Bird Communities across Central Europe
Source: PLoS One. 2014 Nov 11;9(11):e112347. doi: 10.1371/journal.pone.0112347 (PMC4227846; doi:10.1371/journal.pone.0112347)
Supplement: Appendix S2 — Formula used to calculate the “nugget”. (DOCX) [file pone.0112347.s003.docx]

Appendix S2. Formula used to calculate the “nugget.”

Renner, Gossner, Kahl, Kalko, Weisser, et al.

Additional formula in non-linear least squares and convergence of models with *D*’ = dissimilarity and *x* = geographic distance. Formulas are identical to those introduced by Brownstein *et al.* (2012) [17].

| Model | Formula (*D*’) | Nugget | Models  converged | n/a | Negative  nuggets | Nugget  >1 |
| --- | --- | --- | --- | --- | --- | --- |
| Gompertz | *a* • e ^–^*^b •^* ^e ^–^*^c • x^* | *a* • e ^–^*^b^* | 26 | 5 | 0 | 1 |
| Negative exponential | *a* • (1 – *b* • e *^–c • x^*) | *a* • *(1 – b)* | 26 | 4 | 1 | 1 |
| Amended Michaelis-Menten | *a* + *b* • *x* / (*c* + *x*) | *a* | 3 | 0 | 0 | 0 |
| Asymptotic regression | *a* – *b* • *c* ^–^*^x^* | *a – b* | 0 | 0 | 0 | 0 |
| Extreme value | *a* • (1 – e *^b • x + c^*) | *a* • (1 – e *^c^*) | 13 | 0 | 1 | 0 |
| Logistic | *a* / (1 + e *^–b • x + c^*) | *a* / (1 – e *^c^*) | 0 | 0 | 0 | 0 |
