# Supplementary material for: Temporal Changes in Randomness of Bird Communities across Central Europe
Source: PLoS One. 2014 Nov 11;9(11):e112347. doi: 10.1371/journal.pone.0112347 (PMC4227846; doi:10.1371/journal.pone.0112347)
Supplement: Appendix S3 — Summary statistics of GLMM results. (DOCX) [file pone.0112347.s004.docx]

Appendix S3. Summary statistics of GLMM results.

Renner, Gossner, Kahl, Kalko, Weisser, et al.

Testing species richness of birds over five years with effects of year, land-use intensity, and site.

Generalized Linear Mixed Model fit by maximum likelihood, formula: species richness ~ year * region * LUI + (year | site ID)

| Fixed effects | Estimate | Std. Error | z-value | Pr(>\|z\|) | P |
| --- | --- | --- | --- | --- | --- |
| (Intercept) | -123.000 | 68.330 | -1.800 | 0.072 |  |
| year | 0.062 | 0.034 | 1.834 | 0.067 |  |
| explHAI | 193.200 | 86.160 | 2.243 | 0.025 | * |
| explSCH | -53.100 | 94.900 | -0.560 | 0.576 |  |
| lui | 19.900 | 42.830 | 0.465 | 0.642 |  |
| year:explHAI | -0.096 | 0.043 | -2.243 | 0.025 | * |
| year:explSCH | 0.026 | 0.047 | 0.561 | 0.575 |  |
| year:lui | -0.010 | 0.021 | -0.466 | 0.641 |  |
| explHAI:lui | -97.600 | 62.980 | -1.550 | 0.121 |  |
| explSCH:lui | -9.859 | 67.910 | -0.145 | 0.885 |  |
| year:explHAI:lui | 0.049 | 0.031 | 1.551 | 0.121 |  |
| year:explSCH:lui | 0.005 | 0.034 | 0.142 | 0.887 |  |

Generalized Linear Model, formula: species richness ~ year + region + LUI

| Fixed effects | Estimate | Std. Error | z-value | Pr(>\|z\|) | P |
| --- | --- | --- | --- | --- | --- |
| (Intercept) | -82.406 | 16.451 | -5.009 | 0.000 | *** |
| year | 0.042 | 0.008 | 5.156 | 0.000 | *** |
| explHAI | 0.045 | 0.029 | 1.542 | 0.123 |  |
| explSCH | -0.109 | 0.030 | -3.693 | 0.000 | *** |
| lui | -0.073 | 0.019 | -3.838 | 0.000 | *** |

Testing relative abundance of birds over five years with effects of year, land-use intensity, and site. Listed is the output from R. Generalized linear mixed model fit by maximum likelihood, formula: relative abund ~ year * (region + LUI) + (year | site ID)

| Fixed effects | Estimate | Std. Error | z-value | Pr(>\|z\|) | P |
| --- | --- | --- | --- | --- | --- |
| (Intercept) | -178.437 | 45.853 | -3.892 | 0.000 | *** |
| year | 0.090 | 0.023 | 3.950 | 0.000 | *** |
| explHAI | 250.787 | 39.045 | 6.423 | 0.000 | *** |
| explSCH | -28.630 | 40.052 | -0.715 | 0.475 |  |
| lui | -55.933 | 25.179 | -2.221 | 0.026 | * |
| year:explHAI | -0.125 | 0.019 | -6.418 | 0.000 | *** |
| year:explSCH | 0.014 | 0.020 | 0.713 | 0.476 |  |
| year:lui | 0.028 | 0.013 | 2.218 | 0.027 | * |

Significant codes for all tables in Appendix S3: *** *p* ≤ 0.001; ** *p* ≤ 0.01; * *p* ≤ 0.05
